# The rs10830963 Polymorphism of the MTNR1B Gene: Association With Abnormal Glucose, Insulin and C-peptide Kinetics

**DOI:** 10.3389/fendo.2022.868364

**Published:** 2022-06-06

**Authors:** Daniela Vejrazkova, Marketa Vankova, Josef Vcelak, Hana Krejci, Katerina Anderlova, Andrea Tura, Giovanni Pacini, Alena Sumova, Martin Sladek, Bela Bendlova

**Affiliations:** ^1^ Department of Molecular Endocrinology, Institute of Endocrinology, Prague, Czechia; ^2^ Department of Obstetrics and Gynecology, 1st Faculty of Medicine, Charles University and General University Hospital in Prague, Prague, Czechia; ^3^ Metabolic Unit, Institute of Neuroscience, National Research Council, Padova, Italy; ^4^ Laboratory of Biological Rhythms, Institute of Physiology of the Czech Academy of Sciences, Prague, Czechia

**Keywords:** type 2 daibetes mellitus, insulin sensitivity, beta cell function, *MTNR1B* gene, rs10830963, OGTT trajectories, glucose tolerance

## Abstract

**Background:**

The *MTNR1B* gene encodes a receptor for melatonin, a hormone regulating biorhythms. Disruptions in biorhythms contribute to the development of type 2 diabetes mellitus (T2DM). Genetic studies suggest that variability in the *MTNR1B* gene affects T2DM development. Our aim was to compare the distribution of the genetic variant rs10830963 between persons differing in glucose tolerance in a sample of the Czech population (N=1206). We also evaluated possible associations of the polymorphism with insulin sensitivity, beta cell function, with the shape of glucose, insulin and C-peptide trajectories measured 7 times during a 3-hour oral glucose tolerance test (OGTT) and with glucagon response. In a subgroup of 268 volunteers we also evaluated sleep patterns and biorhythm.

**Results:**

13 persons were diagnosed with T2DM, 119 had impaired fasting blood glucose (IFG) and/or impaired glucose tolerance (IGT). 1074 participants showed normal results and formed a control group. A higher frequency of minor allele G was found in the IFG/IGT group in comparison with controls. The GG constellation was present in 23% of diabetics, in 17% of IFG/IGT probands and in 11% of controls. Compared to CC and CG genotypes, GG homozygotes showed higher stimulated glycemia levels during the OGTT. Homozygous as well as heterozygous carriers of the G allele showed lower very early phase of insulin and C-peptide secretion with unchanged insulin sensitivity. These differences remained significant after excluding diabetics and the IFG/IGT group from the analysis. No associations of the genotype with the shape of OGTT-based trajectories, with glucagon or with chronobiological patterns were observed. However, the shape of the trajectories differed significantly between men and women.

**Conclusion:**

In a representative sample of the Czech population, the G allele of the rs10830963 polymorphism is associated with impaired early phase of beta cell function, and this is evident even in healthy individuals.

## Introduction

The *MTNR1B* (melatonin receptor 1B) gene encodes a receptor for melatonin, a hormone that controls biorhythms. The gene is expressed primarily in the brain, but its expression has also been described in pancreatic cells (1). Genetic studies suggest that variability in the *MTNR1B* gene is one of the factors influencing the pathophysiology of type 2 diabetes mellitus (T2DM), with the single nucleotide polymorphism rs10830963 showing the strongest association (2–4).

It has been well documented that melatonin inhibits insulin secretion (5). This inhibition is more pronounced in carriers of the minor variant G of the rs10830963 C/G polymorphism. The reason is that minor variant G confers increased expression of the melatonin receptor in the human pancreas and leads to increased melatonin signaling (5). This poses a higher risk of T2DM in these individuals. The mechanism by which this intronic variant affects enhancer binding, thereby significantly altering gene expression, has already been explained (6). In addition, it has been found that acute melatonin administration leads to impaired glucose tolerance in certain circumstances, depending on the time of administration. Melatonin administered in the morning increased the area under the glycemic curve (AUC) during the OGTT by 186% and maximal glucose concentration during the OGTT by 21% in comparison with placebo administration, while melatonin administered in the evening increased AUC by 54% and maximal glucose concentration by 27% compared to placebo (7). Moreover, this impairment of glucose tolerance was exacerbated in carriers of the minor allele G of the rs10830963 polymorphism. As regards morning administration, the effect of melatonin was six times higher in G allele carriers. In the evening, the effect of melatonin did not differ significantly between G allele carriers and non-carriers (8).

Based on these findings, our aim was to build on our work of 2014 (9) and compare the distribution of the genetic variant rs10830963 between persons differing in glucose tolerance. Subjects with impaired fasting blood glucose (IFG) and/or impaired glucose tolerance (IGT) during a 3-hour oral glucose tolerance test (OGTT) were compared with healthy controls. The genotypes were then assessed in terms of insulin sensitivity (IS), beta cell function, glucagon response, hepatic extraction and other characteristics of glucose metabolism. The novelty of the study lay in the evaluation of possible associations of the polymorphism with the shape of glucose, insulin and C-peptide trajectories (monophasic, biphasic, triphasic, or more complex) formed on the basis of seven measurements during the OGTT, and also with glucagon dynamics (four measurements during the test). There are several current approaches for assessing glucose tolerance, IS and beta cell function *in vivo*. Of these, OGTT and the derived equations (IS and insulin secretion indices) is generally considered the most suitable approach for epidemiological studies. As OGTT provides diagnostic information limited to specific time points, a novel method of monitoring the entire curve has begun to be used to reflect an individual´s metabolic information, such as abnormal IS and impaired insulin secretion. This new method is based on evaluations of the shape of the glucose curve after a fixed oral dose of glucose (10). Glycemia is measured in 30 min intervals during the standard 2-hour OGTT or during the prolonged 3-hour OGTT. In this study, 3-hour trajectories of glucose and also of insulin and C-peptide were monitored with sampling at 0, 30, 60, 90, 120, 150 and 180 min of the test.

It has been postulated that melatonin might influence insulin secretion through a paracrine effect of glucagon (1). Therefore, glucagon was sampled four times at 60 min intervals (0, 60, 120 and 180 min) during the 3-hour OGTT in order to gain a deeper insight into the glucose metabolism of the subjects.

As low first-pass insulin extraction by the liver is considered a risk factor for insulin resistance (11), hepatic extraction was also included in the calculations and analyzed in relation to the variability of the MTNR1B gene.

Melatonin is a hormone that controls sleep and biorhythms and disruption of its natural secretory rhythmicity is considered to be one of the causes of impaired glucose metabolism. Therefore, a pilot study evaluating a possible association of melatonin receptor polymorphism with sleep and chronotype was performed in a subgroup of 268 volunteers.

The whole study was performed on a cohort of the Czech population, for which the rs10830963 polymorphism has not yet been evaluated in detail. Unlike many other European countries, the genographic variability of the Czech population is considerable, because as a country in the middle of Europe it has been affected by many different human migrations that have passed through Europe over time. The highest proportion of Czech people have Slavic origin (approximately 45%), followed by those with Germanic origin (25%) and then Scandinavian and Mediterranean (represented by 7 and 6%, resp.). In short, all study participants represent a combination of Eastern and Western Europeans of Caucasian descent.

## Materials and Methods

### Study Subjects

In the years 2001–2020, adult volunteers with varying degrees of glucose tolerance were continuously examined at the Institute of Endocrinology in Prague. Examinations were based on genetic, anthropometric and biochemical characterization, including the 3-hour OGTT with 75g of glucose load. Exclusion criteria were the presence of serious diseases where passing a glucose test would pose a health risk, and pregnancy, as it is associated with specific changes in glucose metabolism. We also did not include people previously diagnosed with T2DM. Over the 20 years of assembling this cohort, we have used the same protocol and the same or very comparable methods. A total of 1206 volunteers were examined in the study: 985 women (mean age ± SD=32.8 ± 9.35 years, mean BMI ± SD=24.5 ± 5.21 kg/m^2^) and 221 men (mean age ± SD=34.2 ± 11.88 years, mean BMI ± SD=25.1 ± 4.09 kg/m^2^).

### Ethics Approval Statement

The study protocol was in accordance with the institutional ethics committee (Ethics committee of the Institute od Endocrinology EK_EÚ_10062019) and national legislation complying with the principles laid down in the Declaration of Helsinki. Written consent to participate in the study was obtained from all participants.

### Genotyping

DNA was extracted from peripheral leukocytes (QIAamp DNA Blood Kit, QIAGEN, Germany). Genotyping of rs10830963 in the MTNR1B gene was performed using ABI TaqMan SNP Genotyping Assays (LightCycler 480 System, Roche).

### Clinical and Biochemical Characterization

Basic anthropometric characteristics were determined to calculate the body mass index (BMI), waist to hip ratio (WHR), and the body adiposity index (BAI) estimating the amount of total body fat (12).

Venous blood samples were taken at 8 a.m. after overnight fasting. During the 3-hour OGTT (75g of glucose in 250 ml of water), 7 samples were collected in 30 min intervals and blood glucose (enzymatic reference method with hexokinase, Roche), C-peptide (ECLIA, Roche) and insulin concentrations (ECLIA, Roche) were measured. Glucagon (EURIA glucagon radioimmunoassay, EuroDiagnostica AB, Sweden) was measured four times in 60 min intervals (at 0, 60, 120 and 180 min) during the OGTT. The areas under the glycemic (AUC_Glc_), insulin (AUC_Ins_) and C-peptide (AUC_C-pep_) curves were calculated with the trapezoidal rule. Trajectories of blood glucose, insulin and C-peptide were analyzed according to Tura et al. (10). Fasting IS was assessed by HOMA-R and QUICKI, while dynamic IS was evaluated by ISIcomp also known as the Matsuda index, OGIS and PREDIM (13, 14). Beta cell function was evaluated by HOMA-B at fasting state and by the insulinogenic index IGI in dynamics (15). Further indices of beta cell function (in relation to IS) are the disposition index DI (16) computed as OGIS × AUC_Ins_ and the adaptation index AI (17) computed as OGIS × AUC_C-pep_. Hepatic insulin extraction was evaluated according to Tura et al. (18).

### Classification of Glycemic, Insulin and C-peptide Curves During the OGTT

The 3-hour glucose, insulin and C-peptide trajectories were monitored in 30 min intervals (at 0, 30, 60, 90, 120, 150 and 180 min) of the OGTT. The shape of the glucose curve was classified as monophasic when glycemia simply increased and then gradually decreased (one peak). The curve was biphasic when glycemia showed a further increase following the decrease. A three-phase shape was characterized by two complete peaks. In the prolonged 3-hour OGTT, much more complex and heterogeneous curve shapes were observed. In some people, there were also four- and five-phase curves with 3 and 4 complete peaks, respectively. These were classified together as multiphasic glycemic curves. Glucose variations were considered significant if the difference was at least 2% (this criterion was necessary to avoid the detection of false minima and maxima in the glucose curve). For the curves of insulin and C-peptide, criteria with higher requirements for significant variability (5%) were used.

### Chronotype Assessment

Sleep patterns and biorhythms in a pilot subgroup of 268 volunteers were assessed using questions from Munich ChronoType Questionnaire (19) translated into the Czech language. The chronotype was calculated from the mid-sleep phase corrected for sleep debt accumulated during working days, adjusted for age and gender (20). Apart from using sleep phase, circadian preferences were also determined on the basis of self-assessment, asking the subjects to indicate the interval during which they experience the highest cognitive alertness (maximum activity/performance), from which we calculated its midpoint.

### Statistical Analysis

To assess deviations from the Hardy-Weinberg equilibrium of genotype frequencies, the Chi-square test was used. Allele/genotype frequencies were compared between groups by the Chi-square test. Odds ratios and 95% Confidence Intervals were calculated in MedCalc Software. Differences in biochemical and anthropometric data between groups were tested by the non-parametric Mann-Whitney test owing to the non-normal data distribution. The Kruskal-Wallis Z-Value Test with Bonferroni correction was used for multiple comparisons. The power analysis was conducted using the NCSS2020/PASS software. The p-values <0.05 (two tailed) were considered significant.

## Results

### Glucose Metabolism

According to the results of the 3-hour OGTT, participants were divided into three groups: 13 persons were newly diagnosed with T2DM (fasting glycemia ≥7 mmol/l or/and glycemia at 120 min of the test ≥11.1 mmol/l) and formed a T2DM group (21). 119 persons had IFG (fasting glycemia ≥5.6 mmol/l) or IGT (glycemia at 120 min of the test ≥7.8 mmol/l) or these probands met both of the criteria and together they formed an IFG/IGT group. 1074 participants showed normal results (fasting glycemia <5.6 mmol/l and at the same time plasma glucose at 120 min of the test did not rise to 7.8 mmol/l or above) and formed the control group.

### Genotypic Frequencies

The distribution of *MTNR1B* rs10830963 genotypic frequencies did not deviate from Hardy-Weinberg equilibrium (Chi^2 =^ 0.825, p=0.36). The frequency of the minor allele G in the whole cohort was 33.2%. A higher frequency of minor allele G was found in the IFG/IGT group compared to controls (40.7% vs. 32.4%, p=0.01), OR=1.57, CI 95% [1.06; 2.33], p_OR_=0.03). The GG constellation was present in 23% of diabetics, in 17% of the IFG/IGT probands and in 11% of controls (Chi^2 =^ 11.2. p=0.02).

Raw genotype data and complete genotypic and allelic frequencies of our cohort of 1206 participants were published in public repository Figshare.com (22), item. “The rs10830963 SNP of the MTNR1B gene in the Czech cohort” with DOI for public link: 10.6084/m9.figshare.16586039.

### Comparisons of Biochemical Parameters Between the Genotypes

Anthropometric characteristics of the compared genotypic groups are shown in **Table 1**. The female to male ratio was comparable in all genotypic groups. Furthermore, no clinically significant differences between the groups were observed in age, BMI or in body adiposity measured by BAI, or in body fat distribution monitored by WHR. These observations fulfill an important condition for comparing biochemical data.

**Table 1:** Anthropometric characteristics depending on the *MTNR1B* rs10830963 genotype.

	CC (n=545)	CG (n=521)	GG (n=140)	p-level<0.05
Males proportion	103 (19%)	96 (18%)	22 (16%)	ns
Age (years)	32.0 [30.8; 32.5]	32.0 [30.8; 32.5]	31.1 [29.3; 32.9]	ns
Body Weight (kg)	69.3 [67.8; 71.3]	67.3 [64.9; 69.0]	67.9 [63.7; 70.6]	ns
Body Heigh (cm)	170 [169; 171]	169 [168.1; 170]	168.6 [167.6; 170.5]	ns
Waist circumference (cm)	77.7 [76.2; 79.0]	75.6 [74.5; 76.6]	74.9 [73.5; 79.0]	ns
Abdominal circumference (cm)	85.4 [84.2; 86.9]	84.0 [82.3; 85.4]	84.5 [81.5; 87.5]	ns
Hip circumference (cm)	100 [99.1; 101.0]	98.5 [98.0; 99.7]	99 [97.5; 101.0]	CCxCG p=0.01
BMI (kg/m^2^)	23.8 [23.4; 24.4]	23.2 [22.7; 23.6]	23.5 [22.2; 24.6]	ns
WHR	0.78 [0.77; 0.79]	0.77 [0.77; 0.78]	0.77 [0.76; 0.79]	ns
BAI (%)	26.8 [26.3; 27.1]	26.7 [26.3; 27.1]	26.9 [25.5; 28.2]	ns

Data are given as medians [95% LCL; 95% UCL], p-level according to Kruskal-Wallis Z-Value Test with Bonferroni correction.

ns, not significant.

**Figure 1:**
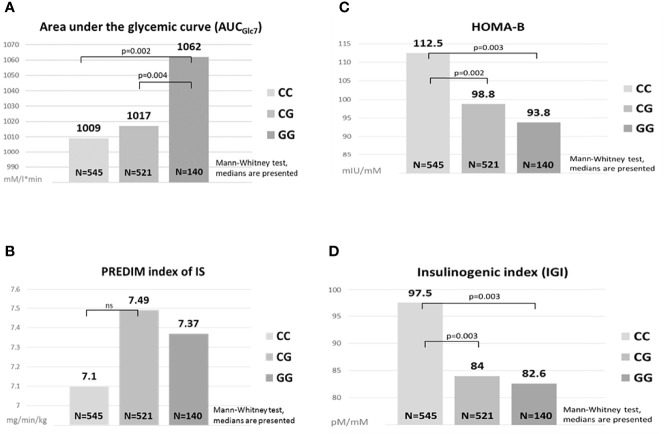
Comparisons of glucose metabolism between the *MTNR1B* genotypes. Area under the glycemic curve **(A)**, HOMA-B **(C)**, PREDIM index of IS **(B)**, Insulinogenic index **(D)**. ns, not significant.

Parameters of glucose metabolism depending on the *MTNR1B* rs10830963 genotype are listed in **Table 2** and **Figure 1**. The GG genotype showed slightly higher basal glycemia compared to the CC genotype. GG homozygotes also showed higher stimulated glycemia (AUC_Glc_) during the OGTT in comparison with CC homozygotes and with heterozygotes (**Figure 1A**). The time points showing the most significant differences were at 30 min (CC vs. GG: p=5x10^-5^, CC vs. CG: p=0.004) and at 60 min (CC vs. GG: p=6x10^-5^, CC vs. CG: p=6x10^-4^), weaker but still highly significant differences were at 90 min of the test (p<0.01 for both comparisons). Insulin sensitivity indices (fasting and dynamic) were not different between the genotype groups (**Table 2** and **Figure 1B**). Homozygous as well as heterozygous carriers of the G allele showed lower HOMA-B and IGI indices of beta cell function in comparison with wild-type CC homozygotes (**Figures 1C, D**), signifying reduced insulin secretion since the hepatic extraction did not differ between the three genotypes (**Table 2**). This assumption strongly supports the observation of the significantly reduced secretion of both insulin and C-peptide at 30 min of the OGTT in G allele carriers compared to the CC genotype (p=0.03 and 0.02, resp.), although the overall 3-hour insulinemia and C-peptidemia measured by AUC_Ins_ and AUC_C-pep_ did not mirror impaired beta cell response. This indicates that the very early phase of insulin secretion is attenuated or delayed in G allele carriers, which then translates into higher blood glucose during the first two hours of the test.

**Table 2:** Parameters of glucose metabolism depending on the *MTNR1B* rs10830963 genotype.

	CC (n=545)	CG (n=521)	GG (n=140)	p-level<0.05
Basal glycemia (mM/l)	4.7 [4.6; 4.7]	4.7 [4.7; 4.8]	4.8 [4.7; 4.9]	CCxGG p=0.01
Basal insulinemia (mIU/l)	6.3 [5.9; 6.5]	5.9 [5.5; 6.2]	5.65 [4.9; 6.4]	ns
AUC_Ins_ (pM x min/l)	33201 [31464; 34893]	31248 [30267; 33489]	35752.5 [31977; 39870]	CGxGG p=0.04
Basal C-peptide (nM/l)	0.61 [0.59; 0.63]	0.57 [0.55; 0.60]	0.57 [0.54; 0.62]	ns
AUC_C-pep_ (pM x min/l)	3.7x10^5^ [3.5x10^5^, 3.8x10^5^]	3.5x10^5^ [3.4x10^5^; 3.7x10^5^]	3.9x10^5^ [3.5x10^5^; 4.2x10^5^]	CGxGG p=0.01
HOMA-R (mIU x mM/l^2^)	1.30 [1.23; 1.36]	1.25 [1.18; 1.32]	1.24 [1.04; 1.36]	ns
HOMA_B (mIU/mM)	112.5 [106.7; 118.2]	98.8 [94.7; 106.2]	93.8 [80.0; 106.7]	CCxCG p=0.002
				CCxGG p=0.003
QUICKI	0.37 [0.36; 0.37]	0.37 [0.37; 0.37]	0.37 [0.36; 0.38]	ns
OGIS_180min_ (ml/min/m^2^)	506.3 [497.9; 511.9]	511.1 [503.7; 517.9]	519.3 [495.5; 534.2]	ns
ISI_COMP_ ([(mg/dl)^2^(μU/ml)^2^]^-1/2^)	8.78 [8.24; 9.20]	8.89 [8.55; 9.35]	8.76 [7.72; 9.48]	ns
Ins_0min_/Glc_0min_ (pM/mM)	8.09 [7.63; 8.50]	7.47 [7.00; 7.96]	7.13 [6.24; 7.60]	CCxCG p=0.04 CCxGG p=0.02
Disposition Index DI	1.7x10^7^ [1.6x10^7^; 1.8x10^7^]	1.6x10^7^ [1.5x10^7^; 1.7x10^7^]	1.8x10^7^ [1.6x10^7^; 2.0x10^7^]	CGxGG p=0.04
Adaptation Index AI	1.9x10^8^ [1.8x10^8^; 1.9x10^8^]	1.8x10^8^ [1.8x10^8^; 1.9x10^8^]	1.9x10^8^ [1.9x10^8^; 2.1x10^8^]	CGxGG p=0.01
Hepatic insulin extraction (%)	69.2 [68.2; 69.8]	70.1 [69.2; 70.9]	69.5 [67.1; 70.9]	ns
Basal glucagonemia (pM/l)	36.5 [35.2; 37.5]	37.05 [36.5; 37.9]	37.5 [34.8; 40.2]	ns

Data are given as medians [95% LCL; 95% UCL], p-level according to Mann-Whitney test.

ns, not significant.

The differences observed between the genotypes in AUC_Glc_ and in the indices of beta cell function (HOMA-B, IGI) remained statistically significant after excluding diabetics from the analysis. Moreover, all the described differences remained fully preserved even after the exclusion of the IFG/IGT group, which indicates the effect of the G allele in completely healthy individuals in terms of glucose control. Within the IFG/IGT group alone, the effect of the allele was systematically evident, but as this group was less numerous compared to controls, the differences were not significant.

Evaluations of the relationship between the polymorphism and glucagon levels showed no association with either fasting glucagon or post-glucose glucagon response at 60, 120 or 180 min of the OGTT. In addition, no association of the genotype with hepatic extraction was observed in either the overall cohort or in any of the subgroups.

### Comparisons of Glycemic, Insulin and C-peptide Curves Between the Genotypes

Analysis of the *MTNR1B* rs10830963 in relation with trajectories of glucose, insulin and C-peptide showed that the three genotypes are distributed equally within the four different types of curves (monophasic, biphasic, triphasic, or more complex), **Table 3**.

**Table 3:** Proportions of the *MTNR1B* rs10830963 genotype depending on the shape of glycemic, insulinemic and C-peptide curves.

MTNR1B genotype (%)				STATISTICS
**Glycemic curve**	**CC**	**CG**	**GG**	
monophasic	48	45.5	56	Chi^2 =^ 5.9
biphasic	21	22.5	16.5	power=0.40
triphasic	26	26	21.5	p-level=0.43
multiphasic	5	6	6	
**Insulinemic curve**	**CC**	**CG**	**GG**	
monophasic	69.5	67	72	Chi^2 =^ 1.85
biphasic	6.5	6.5	6	power=0.14
triphasic	22	24.5	20	p-level=0.93
multiphasic	2	2	2	
**C-peptide curve**	**CC**	**CG**	**GG**	
monophasic	80	77	81	Chi^2 =^ 5.11
biphasic	1	2	1	power=0.34
triphasic	18	20	18	p-level=0.53
multiphasic	1	1	0	

However, a different distribution of men and women was observed in the particular categories of glycemic curves, **Table 4**. Significantly more men had a biphasic curve (the percentage of men showing biphasic glucose response was double that of women), and significantly more women had a triphasic curve. This finding was independent of genotype, the difference between the genders was significant within all three genotypes, as shown in **Table 5**.

**Table 4:** Gender proportions depending on the shape of glycemic curves.

Gender proportion (%)			Statistics
Glycemic curve	Females	Males	
monophasic	49	41	Chi^2 =^ 63.4
biphasic	17	40	power=1.00
triphasic	28	14	**p-level<0.000001**
multiphasic	6	5	

Statistically significant results are in bold.

**Table 5:** Gender proportions depending on the *MTNR1B* rs10830963 genotype and on the shape of glycemic curves.

Gender proportions			STATISTICS
**Gender proportion in CC homozygotes (%)**			
**Glycemic curve**	**Females**	**Males**	
monophasic	49	42	Chi^2 =^ 39.8
biphasic	16	43	power=1.00
triphasic	29	12	**p-level=<0.000001**
multiphasic	6	3	
**Gender proportion in CG heterozygotes (%)**			
**Glycemic curve**	**Females**	**Males**	
monophasic	47	39	Chi^2 =^ 18.8
biphasic	18	39	power=0.97
triphasic	28	18	**p-level=0.0003**
multiphasic	7	4	
**Gender proportion in GG homozygotes (%)**			
**Glycemic curve**	**Females**	**Males**	
monophasic	59	36	Chi^2 =^ 10.9
biphasic	13	36	power=0.80
triphasic	23	14	**p-level=0.01**
multiphasic	5	14	

Statistically significant results are in bold.

Graphs and tables showing medians of glucose, insulin, C-peptide as well as glucagon dynamics at all time points measured during the OGTT for each genotype are available in the **Supplementary Material A**.

### Comparisons of Sleep Regime and Chronotype Between the Genotypes

In the subcohort of 268 volunteers who completed the questionnaire data for this pilot study, minor variant G was present in a heterozygous constellation in 124 participants (46%) and in a homozygous constellation in 26 (10%) with an allelic frequency of 33%. The remaining 118 individuals (44%) were homozygous in the common variant C. The average age and the ratio of women/men did not differ significantly between the compared genotype groups. The average duration of sleep on weekdays and days off did not differ between the genotypes, nor did the mid-sleep phase on weekdays and days off. The chronotype calculated from the mid-sleep phase corrected for sleep debt accumulated during working days and adjusted for age and gender was also comparable. The time of subjective maximum daily activity and performance (best alertness midpoint) was similar in all three genotype groups, with a median at 11 a.m. The social jet lag resulting from the discrepancy between the natural biorhythm and work/social duties averaged 0.85 ± 0.698 h regardless of genotype. Graphs showing medians of sleep and biorhythm patterns for each genotype of the MTNR1B rs10830963 SNP are available in the **Supplementary Material B**.

## Discussion

Meta analyses have indicated relationships between the rs10830963 minor allele G and T2DM, with the G allele associated with higher fasting blood glucose levels in Cuacasians and Asians (23–27). However, limited cross-ethnicity has been observed as regards the effect of the allele on insulin sensitivity, beta cell function (28–31), or whether a minor allele dose effect is apparent. A dose effect of the G allele on the ability of beta cells to maintain blood glucose levels was described in a recent study conducted on almost 190 thousand participants of European descent, and each additional risk allele was associated with 10% higher odds of T2DM (32). A genome-wide association study evaluating IVGTT-based measures of first-phase insulin secretion revealed a strong association of the G allele with a lower first-phase insulin response and also with insulin secretion rate in several different ethnic groups (29). Also, previous data from the OGTT-based Botnia Study showed that the G variant of *MTNR1B* has the strongest effect on beta cell function in nondiabetic participants from the Botnia region of western Finland (30). Accordingly, our data demonstrate lower beta cell function assessed by HOMA-B and IGI indices in homozygous and heterozygous carriers of the G allele. This impairment was not detected by overall 3-hour AUC_Ins_ or AUC_C-pep_, as only data at 30 min directly demonstrated that carriers of the G allele show a reduced secretion of both insulin and C-peptide. During the rest of the OGTT, this effect was no longer noticeable. On the contrary, increasing glycemia led to a gradual compensatory increase in insulin secretion starting with the second hour of the test. It can only be assumed that even more significant differences in insulin secretion between the genotypes would be detected at 15 min. Thus, the early phase of the pancreatic beta cell response to a glucose stimulus, which is attenuated or delayed in G allele carriers, is likely crucial for understanding and interpreting our results. To this end, we are currently adjusting the examination protocol, to evaluate glycemia, insulinemia and C-peptide levels at the 15 min of the OGTT.

The dominant effect of the G allele observed in beta cell function was not apparent in insulin sensitivity. Based on values of the PREDIM index, considered a valuable index due to the close correlation with the clamp method, IS was not reduced in homozygous or heterozygous G allele carriers. In general, beta cell function is the actual discriminant between the genotype groups, as demonstrated by the HOMA-B, IGI, DI and AI indices. This leads to a conclusion useful for clinical practice, supported by the results of previous studies (33, 34). Individuals carrying the G allele could benefit greatly from adjusting their lifestyle so that they are not forced to have breakfast early in the morning, when their melatonin levels are still high, due to work and social responsibilities. These people have a delayed morning drop in melatonin (34). High levels of melatonin disrupt insulin secretion. In addition, G allele carriers are significantly more vulnerable due to increased melatonin signaling (5) and impaired early insulin secretion capacity, and the long-term regular need for insulin secretion due to early morning food intake could expose this group to a greater risk of developing glucose tolerance disorders. The already mentioned shift in melatonin secretion towards a later rise in the evening and a slower decline in the morning in G allele carriers led us to the idea of testing whether the sleep regime and the setting of the entire chronotype are not shifted as well. The results of the pilot study do not yet indicate this, which may be due to the relatively small number of individuals involved. We will continue to test this hypothesis on larger groups.

One of the main benefits of this study is the original data from the Czech population analyzed in a representative cohort that is unique in its size and in the detailed biochemical examinations in the Czech Republic. We comprehensively assessed relationships between the genetic variant in the melatonin receptor and glucose metabolism using both standard and novel indices of inulin sensitivity and beta cell function, as well as by C-peptide and glucagon dynamics during a prolonged OGTT. All the conclusions are based on robust non-parametric evaluations. Innovative is the evaluation of the genetic variant in relation to the shapes of the glucose, insulin and C-peptide trajectories based on sampling before the glucose load and then at 30, 60, 90, 120, 150 and 180 min after it, as studies based on a two-hour OGTT have limited potential to evaluate the shape of the curves. Nevertheless, despite thorough analysis, no effect of the SNP on the shape of the trajectories was apparent. Interestingly, however, we found that twice the percentage of men had a biphasic glycemic curve during the 3-hour OGTT, while a triphasic curve was significantly more common in women. This clearly shows that gender should always be taken into account when evaluating the shapes of glucose trajectories and other related parameters during the OGTT. In this context, the question arises as to whether the blood glucose levels at 120 min of the OGTT represent the optimal criterion for impaired glucose tolerance (21) for both genders, as the different shape of the glycemic trajectory in women and men may require a distinct approach. This issue has not been elaborated in detail in the literature and will be the subject of our research in the future.

One disadvantage of our study was the significantly lower number of individuals with impaired glycemic control compared to healthy controls. The relatively low average age of the participants also contributes to this disparity. However, while maintaining the current longitudinal character of our research, which has been going on for over 20 years, it will be possible to verify existing data on significantly older participants, in whom the proportion of people with glucose metabolism disorders will be significantly higher. Furthermore, a lower proportion of men compared to women can also be considered a weakness. Although we addressed a similar number of men as women, their willingness to participate in clinical trials was significantly lower. However, the gender ratio did not differ in the compared genotype groups, so the impact of this imbalance on the study results is minimized.

In a representative sample of the Czech population, we demonstrated the association of the minor allele G of the rs10830963 polymorphism in the *MTNR1B* gene with glucose metabolism. The G allele was more frequent in people with impaired glucoregulation. Homozygous carriers of this allele showed higher blood glucose levels during the OGTT. Since there were no differences in insulin sensitivity between the genotypes, the higher glycemia was due to lower beta cell function, especially early insulin secretion, observed in homozygous as well as in heterozygous G allele carriers. This association with impaired early pancreatic function was significant even in individuals with healthy glucose processing. As such, the G allele is a factor that may, under certain circumstances, promote higher glucose levels and contribute to the development of glucose intolerance.

## Data Availability Statement

The datasets presented in this study can be found in online repositories. The names of the repository/repositories and accession number(s) can be found in the article/**Supplementary Material**.

## Ethics Statement

The studies involving human participants were reviewed and approved by Ethics committee of the Institute of Endocrinology EK_EÚ_10062019. The patients/participants provided their written informed consent to participate in this study.

## Author Contributions

Conceptualization and design of the work, formal analysis, project administration, original draft preparation and writing: DV. Review & editing, statistical analysis: MV. Methodology of genetic analyzes: JV. Significant data completion: HK, KA. Data curation, indices calculation: GP, AT. Final approval of the work: BB. Processing of chronotype questionnaires: AS, MS. All authors contributed to the article and approved the submitted version.

## Funding

The study was supported by Ministry of Health of the Czech Republic, grant NU20-01-00308. All rights reserved.

## Conflict of Interest

The authors declare that the research was conducted in the absence of any commercial or financial relationships that could be construed as a potential conflict of interest.

## Publisher’s Note

All claims expressed in this article are solely those of the authors and do not necessarily represent those of their affiliated organizations, or those of the publisher, the editors and the reviewers. Any product that may be evaluated in this article, or claim that may be made by its manufacturer, is not guaranteed or endorsed by the publisher.
