# Supplementary material for: The rs10830963 Polymorphism of the MTNR1B Gene: Association With Abnormal Glucose, Insulin and C-peptide Kinetics
Source: Front Endocrinol (Lausanne). 2022 Jun 6;13:868364. doi: 10.3389/fendo.2022.868364 (PMC9207528; doi:10.3389/fendo.2022.868364)
Supplement: Supplementary file 1 [file DataSheet_1.pdf]

Supplementary Material A:

Graphs and tables showing medians of glucose, insulin, C-peptide and glucagon levels at all time points measured during the OGTT for each genotype of the MTNR1B rs10830963 SNP in the whole cohort of 1206 volunteers. N(CC)=545, N(CG)=521, N(GG)=140.

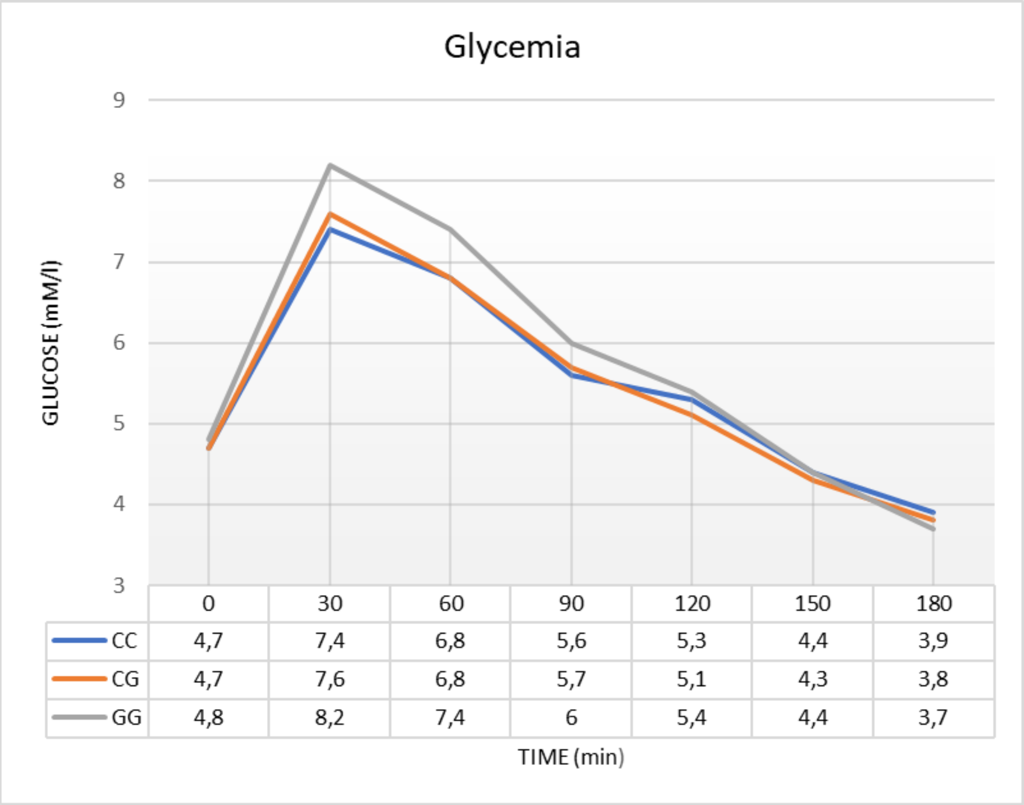

**p-level**	0 min	30 min	60 min	90 min	120 min	150 min	180 min
CCxCG	0,05	**0,04**	0,28	0,94	0,66	0,38	0,20
CCxGG	**0,01**	**0,0001**	**0,0001**	**0,002**	0,28	0,71	0,06
CGxGG	0,15	**0,004**	**0,0006**	**0,003**	0,16	0,36	0,30
p-level according to Mann-Whitney test, significant differences in bold							

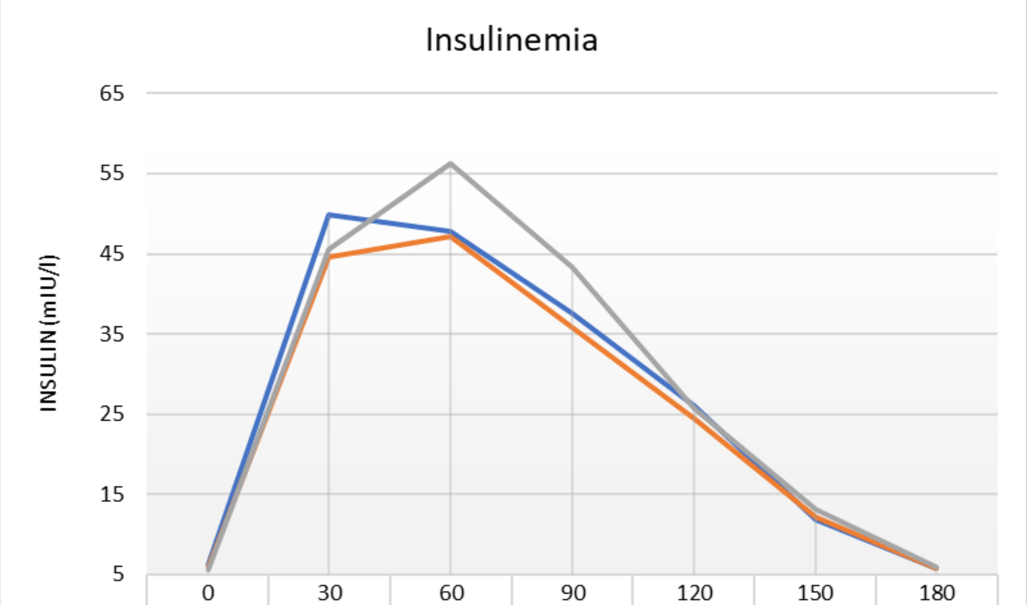

	0	30	60	90	120	150	180
CC	6,3	49,8	47,8	37,5	26,1	11,8	5,7
CG	5,9	44,6	47,1	35,8	24,5	12,1	5,7
GG	5,65	45,5	56,3	43,3	25,75	13,05	5,95

TIME (min)

**p-level**	0 min	30 min	60 min	90 min	120 min	150 min	180 min
CCxCG	0,09	**0,03**	0,70	0,64	0,43	0,83	0,99
CCxGG	0,08	0,33	**0,01**	**0,02**	0,40	0,32	0,20
CGxGG	0,49	0,70	**0,01**	**0,01**	0,18	0,38	0,21

p-level according to Mann-Whitney test, significant differences in bold

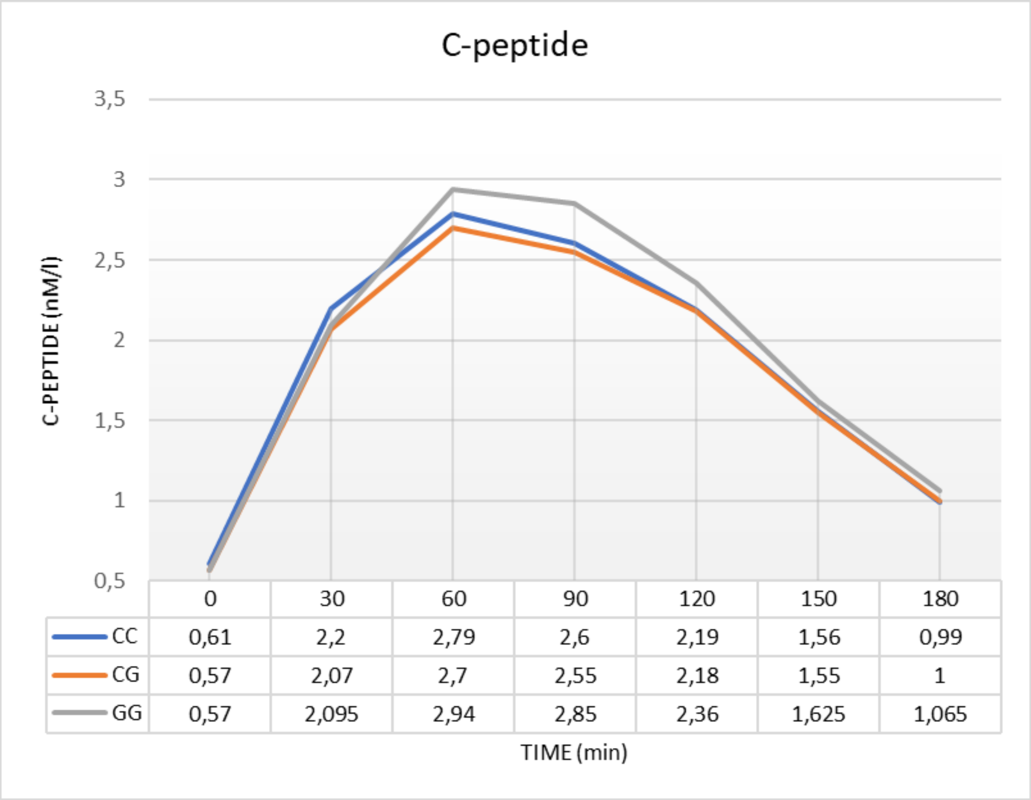

**p-level**	0 min	30 min	60 min	90 min	120 min	150 min	180 min
CCxCG	0,10	**0,02**	0,24	0,50	0,80	0,85	0,81
CCxGG	0,60	0,30	0,05	**0,01**	0,08	0,14	0,05
CGxGG	0,61	0,61	**0,01**	**0,002**	0,05	0,10	0,05

p-level according to Mann-Whitney test, significant differences in bold

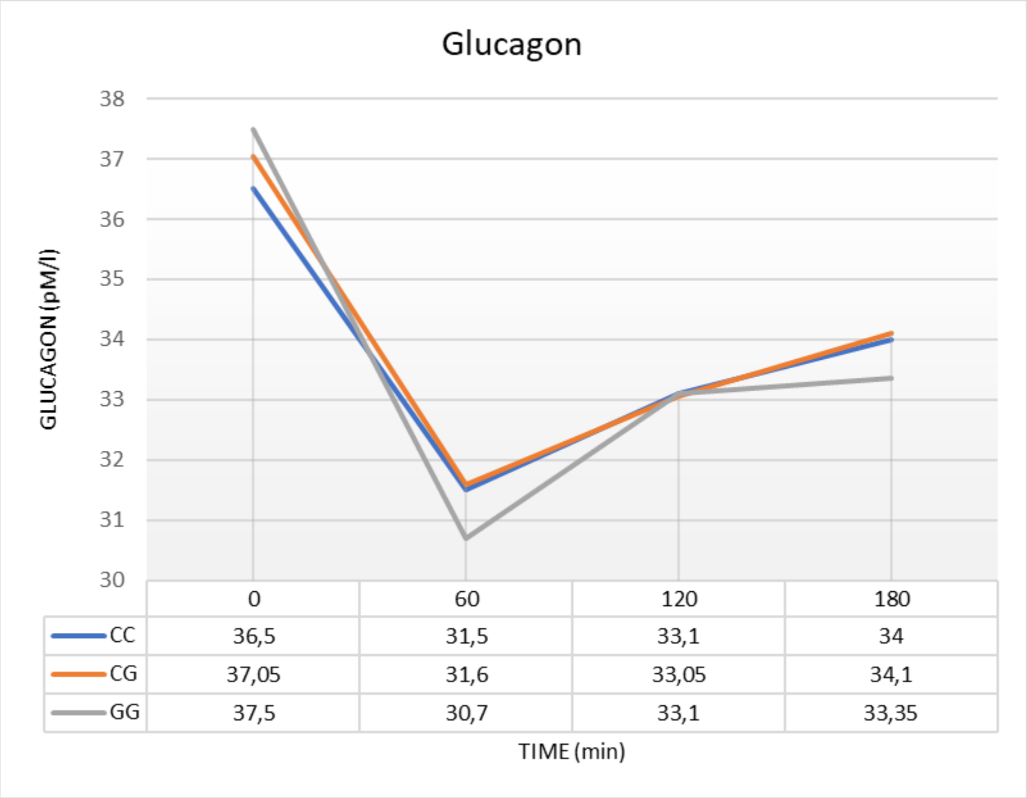

**p-level**	0 min	60 min	120 min	180 min
CCxCG	0,18	0,34	0,61	0,40
CCxGG	0,13	0,94	0,87	0,58
CGxGG	0,54	0,60	0,86	0,91
p-level according to Mann-Whitney test				
