# Supplementary material for: The rs10830963 Polymorphism of the MTNR1B Gene: Association With Abnormal Glucose, Insulin and C-peptide Kinetics
Source: Front Endocrinol (Lausanne). 2022 Jun 6;13:868364. doi: 10.3389/fendo.2022.868364 (PMC9207528; doi:10.3389/fendo.2022.868364)
Supplement: Supplementary file 2 [file DataSheet_2.pdf]

Supplementary Material B:

Graphs showing medians of sleep and biorhythm patterns for each genotype of the MTNR1B rs10830963 SNP in the subcohort of 268 volunteers. N(CC)=118, N(CG)=124, N(GG)=26.

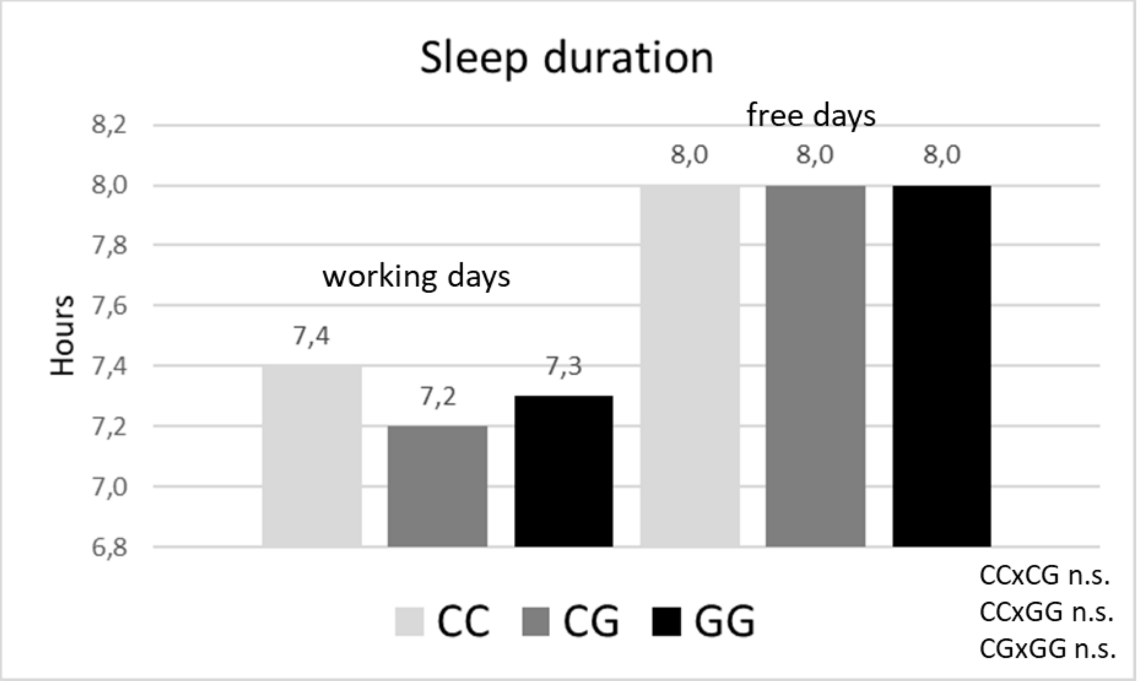

n.s. not significant according to Mann-Whitney test

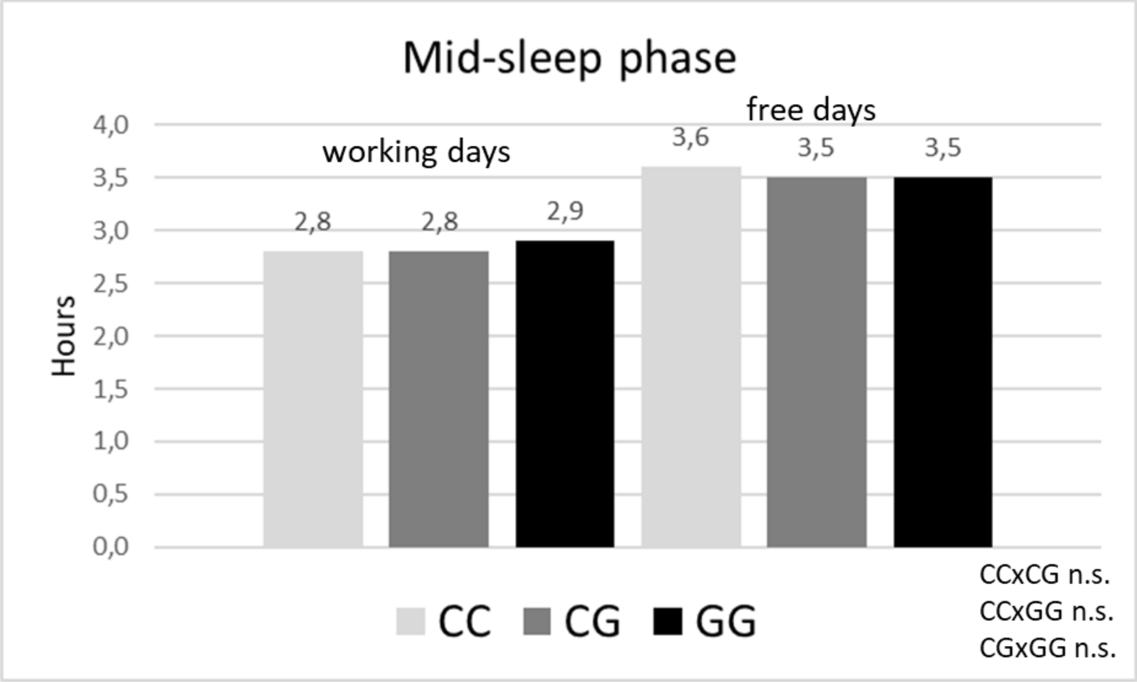

n.s. not significant according to Mann-Whitney test

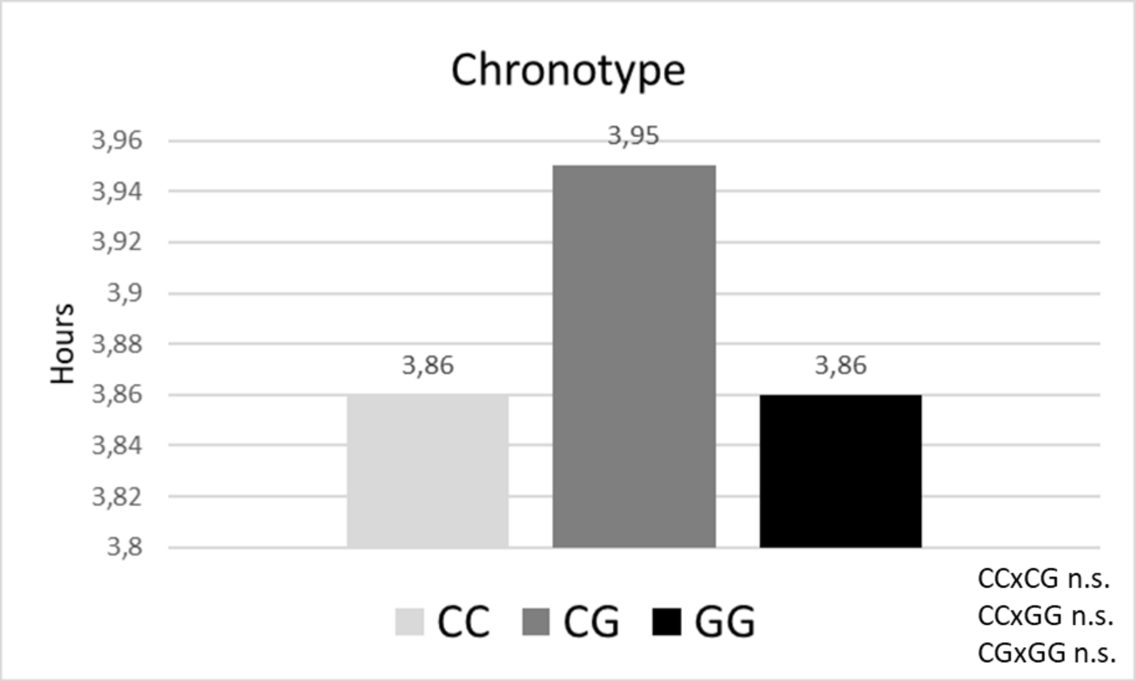

n.s. not significant according to Mann-Whitney test

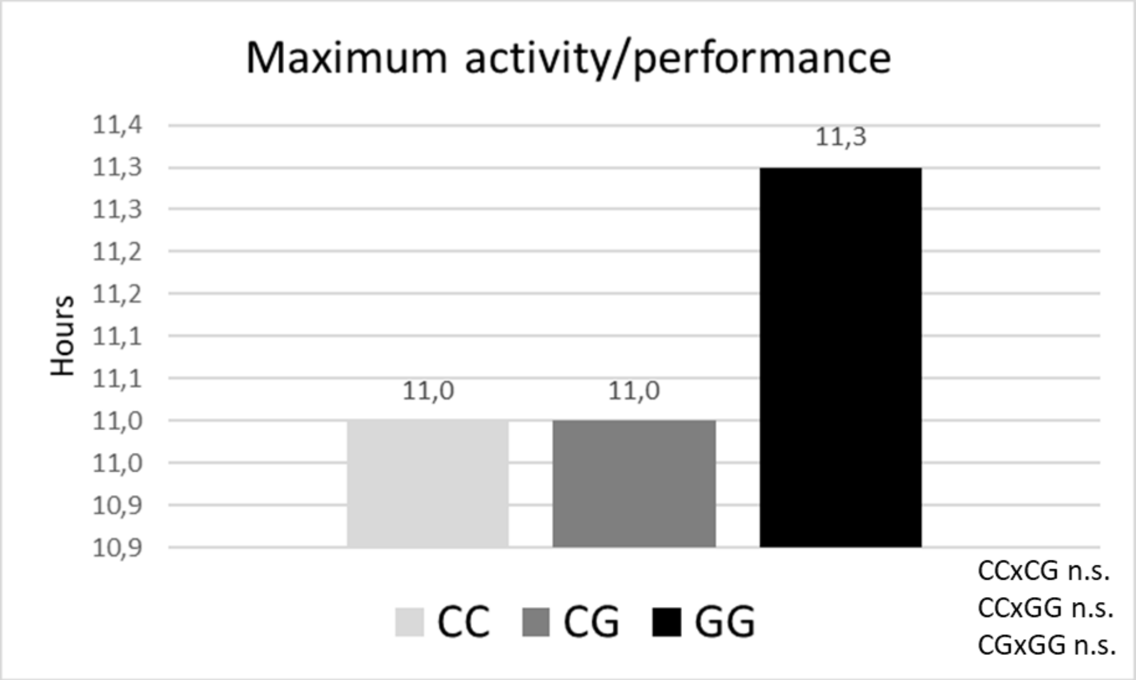

n.s. not significant according to Mann-Whitney test

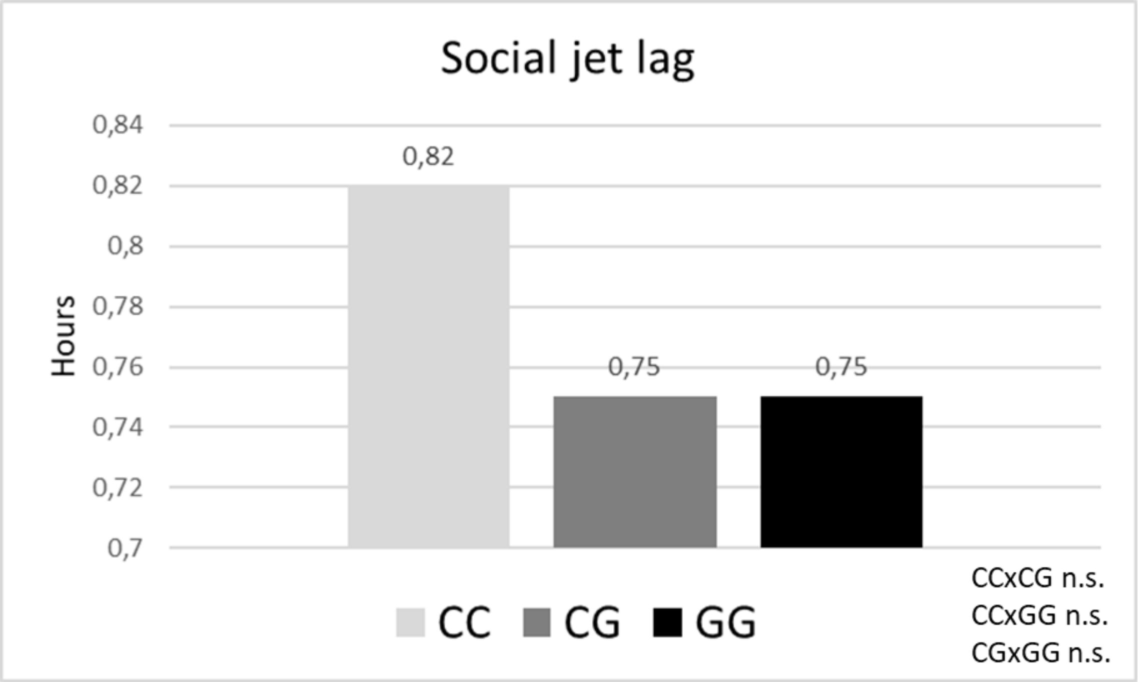

n.s. not significant according to Mann-Whitney test
